# Tumeur métastatique de l'iris et du corps ciliaire d'un cancer bronchopulmonaire

**DOI:** 10.11604/pamj.2015.20.417.6799

**Published:** 2015-04-28

**Authors:** Meriem Abdellaoui, Hicham Tahri

**Affiliations:** 1 Faculté de Médecine et de Pharmacie de Fès, Service d'Ophtalmologie, CHU Hassan II, Fès, Maroc

**Keywords:** Métastase de l′iris, métastase du corps ciliaire, cancer broncho-pulmonaire, Metastasis of the iris, Metastasis of the ciliary body, lung cancer

## Image en medicine

Mme B.N, 39 ans, sans antécédents pathologiques particuliers, consulte dans notre formation pour un œil rouge et douloureux avec baisse de l'acuité visuelle récente du côté gauche. L'acuité visuelle corrigée à gauche est de 4/10^e^. L'examen à la lampe à fente montre un processus tumoral en dôme de coloration jaunâtre et envahissant la racine de l'iris en temporal inférieur (A, B), siégeant au niveau du corps ciliaire inférieur (C). Une fine réaction cellulaire en regard de la tumeur ciliaire (D) et dans la chambre antérieure, est notée. Le tonus oculaire, le reste du segment postérieur, l'examen de l’œil adelphe et l'examen général sont normaux. Ce processus prend modérément le gadolinium à l'IRM. Le bilan d'extension révèle une tumeur pulmonaire dont la biopsie met en évidence un adénocarcinome pulmonaire moyennement différencié. Ainsi le diagnostic d'une métastase de l'iris et du corps ciliaire d'une tumeur pulmonaire est retenu. Après la chimiothérapie, l’évolution est marquée par la régression importante de la tumeur et de sa métastase oculaire à 6 mois, mais aux dépens d'une perte fonctionnelle de cet œil par glaucome secondaire.

**Figure 1:**
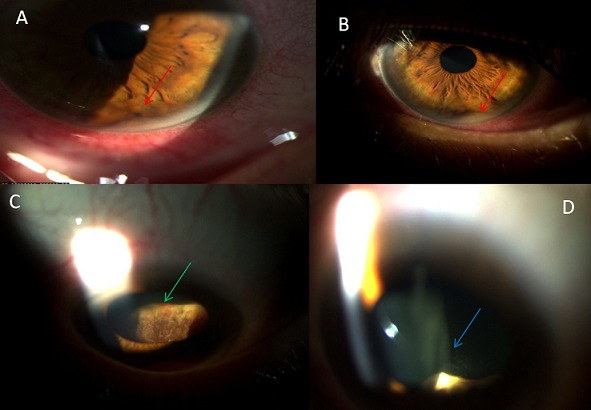
A, B) photographie en lampe à fente montrant la lésion tumorale au niveau de la racine de l'iris (flèche rouge); C) photographie en lampe à fente montrant un processus tumoral en dôme au niveau du corps ciliaire (flèche verte); D) photographie en lampe à fente montrant l'effet tyndall vitréen (flèche bleue) en rapport avec la réaction cellulaire en regard de la lésion ciliaire

